# β-cell–selective inhibition of DNA damage response signaling by nitric oxide is associated with an attenuation in glucose uptake

**DOI:** 10.1016/j.jbc.2023.102994

**Published:** 2023-02-10

**Authors:** Chay Teng Yeo, Erin M. Kropp, Polly A. Hansen, Michael Pereckas, Bryndon J. Oleson, Aaron Naatz, Jennifer S. Stancill, Kyle A. Ross, Rebekah L. Gundry, John A. Corbett

**Affiliations:** Department of Biochemistry, Medical College of Wisconsin, Milwaukee, Wisconsin, USA

**Keywords:** β-cell, nitric oxide, islet, glucose uptake, DDR signaling, metabolism, 2-DG, 2-deoxyglucose, ATM, ataxia–telangiectasia mutated protein, ATR, ATM- and Rad3-related protein, Chk, checkpoint kinase, CMRL, Connaught Medical Research Laboratories, DDR, DNA damage response, DPTA, (Z)-1-[N-(3-aminopropyl)-N-(3-ammoniopropyl)amino]diazen-1-ium-1,2-diolate, ECAR, extracellular acidification rate, ETC, electron transport chain, FACS, fluorescence-activated cell sorting, FCCP, carbonyl cyanide p-trifluoro-methoxyphenyl hydrazine, FoxO1, Forkhead Box O1, H2AX, H2A histone family member X, HRP, horseradish peroxidase, IL-1, interleukin-1, iNOS, inducible isoform of nitric oxide synthase, KAP, Krüppel-associated box-associated protein, LDH, lactate dehydrogenase, MCT, monocarboxylic acid transporter, MEF, mouse embryonic fibroblasts, NO, nitric oxide, OCR, oxygen consumption rate, PARP, poly(ADP-ribose) polymerase, PPP, pentose phosphate pathway, Sirt, sirtuin, TCA, tricarboxylic acid

## Abstract

Nitric oxide (NO) plays a dual role in regulating DNA damage response (DDR) signaling in pancreatic β-cells. As a genotoxic agent, NO activates two types of DDR signaling; however, when produced at micromolar levels by the inducible isoform of NO synthase, NO inhibits DDR signaling and DDR-induced apoptosis in a β-cell–selective manner. DDR signaling inhibition by NO correlates with mitochondrial oxidative metabolism inhibition and decreases in ATP and NAD^+^. Unlike most cell types, β-cells do not compensate for impaired mitochondrial oxidation by increasing glycolytic flux, and this metabolic inflexibility leads to a decrease in ATP and NAD^+^. Here, we used multiple analytical approaches to determine changes in intermediary metabolites in β-cells and non–β-cells treated with NO or complex I inhibitor rotenone. In addition to ATP and NAD^+^, glycolytic and tricarboxylic acid cycle intermediates as well as NADPH are significantly decreased in β-cells treated with NO or rotenone. Consistent with glucose-6-phosphate residing at the metabolic branchpoint for glycolysis and the pentose phosphate pathway (NADPH), we show that mitochondrial oxidation inhibitors limit glucose uptake in a β-cell–selective manner. Our findings indicate that the β-cell–selective inhibition of DDR signaling by NO is associated with a decrease in ATP to levels that fall significantly below the *K*_*M*_ for ATP of glucokinase (glucose uptake) and suggest that this action places the β-cell in a state of suspended animation where it is metabolically inert until NO is removed, and metabolic function can be restored.

The DNA damage response (DDR) is comprised of three kinases that belong to the family of PI3K-related kinases (1, 2). In response to double-strand DNA breaks, ataxia–telangiectasia mutated protein (ATM) and DNA-dependent protein kinase are activated, whereas ATM- and Rad3-related protein (ATR) responds to a broader range of DNA damage such as replication stress and single-strand DNA breaks (2). Once activated, transducer kinases phosphorylate downstream effector proteins, leading to multiple physiological responses that include DNA repair, cell cycle arrest, cellular senescence, or apoptosis if the damage is beyond repair (1, 3).

Pancreatic β-cells play a primary role in the regulation of whole-body glucose homeostasis through the synthesis and secretion of insulin. When exposed to inflammatory cytokines such as interleukin-1 (IL-1), β-cells express the inducible isoform of nitric oxide (NO) synthase (iNOS) and generate micromolar levels of NO. NO mediates the inhibitory actions of cytokines on insulin secretion, oxidative metabolism, protein synthesis, and induces DNA damage (4, 5, 6). Based on these inhibitory and destructive actions, cytokines and NO have been implicated in the loss of functional β-cell mass during the development of diabetes (4, 5, 6). In exploring the response of β-cells to DNA damage, we made the unexpected observation that NO, when generated at iNOS-derived levels (7, 8), has a dual action on DDR signaling that is cell type–selective (9, 10, 11, 12). In all cell types examined to date, NO activates ATM-dependent DDR signaling *via* the induction of DNA-strand breaks and ATR-dependent DDR signaling by inhibiting ribonucleotide reductase (9, 12). Surprisingly, when produced or provided at iNOS-derived low micromolar levels, NO inhibits ATM- and ATR-dependent DDR signaling in a β-cell selective manner (10, 12). In fact, NO limits DDR signaling in the presence of persistent DNA damage and attenuates DDR-dependent β-cell apoptosis, while stimulating DDR signaling and DDR-dependent apoptosis in all other cell types examined to date (10). While cytokines like IL-1 have been implicated in the loss of functional β-cell mass during the induction of diabetes (13), the inhibition of DDR signaling and attenuation of DDR-directed apoptosis suggests that IL-1 signaling may play physiological roles in protecting β-cells from DDR-mediated apoptosis.

One of the well-known actions of iNOS-derived levels of NO is the inhibition of mitochondrial oxidative metabolism. NO inhibits the tricarboxylic acid (TCA) cycle enzyme aconitase and complex IV of the electron transport chain (ETC) (14, 15). The inhibitory actions of NO on mitochondrial metabolism are not cell type selective, but the cellular response to this inhibition is cell type selective (12, 16). Most cell types have the metabolic flexibility to increase glycolytic flux when mitochondrial oxidation is inhibited (17, 18); however, glycolytic and mitochondrial oxidative metabolism are coupled in β-cells as almost all the carbons of glucose are oxidized to CO_2_ in a concentration-dependent manner (19, 20, 21). Because of this coupling, β-cells lack the flexibility to compensate for impaired mitochondrial oxidation with an increase in glycolysis (12, 16). This lack of metabolic flexibility contributes to the β-cell–selective inhibition of DDR signaling by NO (12, 16). Classical inhibitors of mitochondrial respiration such as rotenone (complex I), antimycin A (complex III), and carbonyl cyanide p-trifluoro-methoxyphenyl hydrazine (FCCP) (uncoupler) also attenuate DDR signaling in a β-cell–selective manner (12, 16).

In this study, targeted metabolomic analyses were used to identify potential metabolites and/or metabolic pathways that contribute to the β-cell–selective inhibition of DDR signaling by NO and inhibitors of mitochondrial oxidative metabolism. We show that NO and rotenone decrease the levels of ATP, NAD^+^, NADH, NADPH, several glycolytic intermediates, and citrate in β-cells, whereas the levels of these metabolites are not modified in non–β-cells. Because metabolites in glycolysis and the pentose phosphate pathway (PPP) are decreased in a β-cell–selective manner, we hypothesized that metabolic events upstream of the formation of glucose-6-phosphate, which resides at the branchpoint between glycolysis and the PPP, are decreased in β-cells in response to NO and rotenone. The first step in glucose metabolism is its phosphorylation by hexokinase (most non–β-cells) and glucokinase (β-cells) (22). In this study, we show that the cell type–selective decrease in nucleotides such as ATP, NAD^+^, and NADPH and the inhibition of DDR signaling are associated with the inhibition of glucose uptake in a β-cell–selective manner.

## Results

### A lack of metabolic flexibility is associated with the β-cell–selective inhibition of DDR signaling by NO

**Figure 1:**
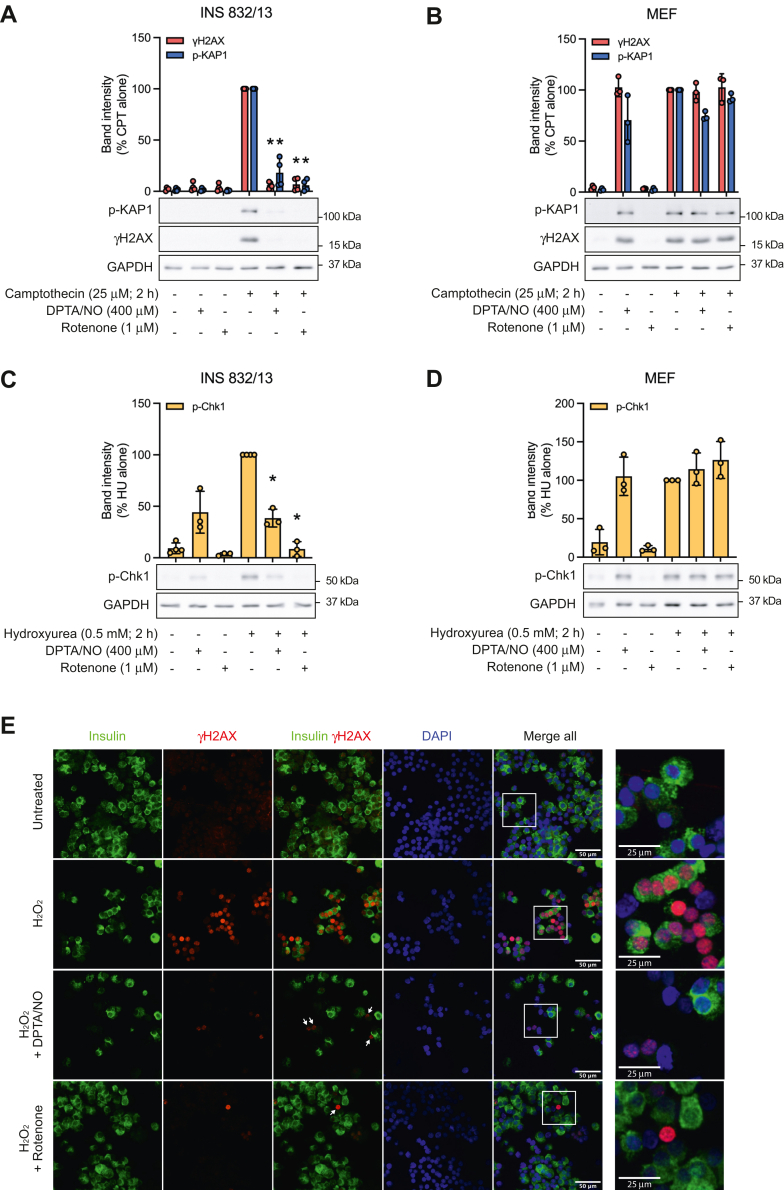
**Nitric oxide (NO) and rotenone inhibit DDR signaling in a β-cell–selective manner.** INS 832/13 cells (*A* and *C*), MEF (*B* and *D*), and dispersed rat islet cells (*E*) were treated with camptothecin (CPT) (*A* and *B*), hydroxyurea (HU) (*C* and *D*), or hydrogen peroxide (H_2_O_2_; 30 min treatment at 100 μM) (*E*) with or without DPTA/NO or rotenone for 2 h at the indicated concentrations. Phosphorylation of H2AX (γH2AX), KAP1, and Chk1 was determined by Western blot analysis, and GAPDH was determined as a loading control (*A*–*D*). Rat islet cells were treated as outlined above (*E*), plated onto slides, fixed, and then were stained for insulin (*green*), γH2AX (*red*), and nuclei (DAPI, *blue*). Images were visualized using a Nikon Eclipse 90i confocal microscope (60× with 2× field zoom). Results are representative of three to four independent experiments. Chk1, checkpoint kinase 1; DAPI, 4′,6-diamidino-2-phenylindole; DDR, DNA damage response; DPTA, (Z)-1-[*N*-(3-aminopropyl)-*N*-(3-ammoniopropyl)amino]diazen-1-ium-1,2-diolate; H2AX, H2A histone family member X; KAP1, Krüppel-associated box-associated protein; MEF, mouse embryonic fibroblasts.

In response to the topoisomerase inhibitor camptothecin, ATM is activated and phosphorylates H2A histone family member X (H2AX) (γH2AX when phosphorylated) and Krüppel-associated box-associated protein (KAP)1 in INS 832/13 cells and mouse embryonic fibroblasts (MEF) (Fig. 1, *A* and *B*). Consistent with our previous findings (10, 12, 16, 23), NO, supplied by the donor (Z)-1-[*N*-(3-aminopropyl)-*N*-(3-ammoniopropyl)amino]diazen-1-ium-1,2-diolate (DPTA)/NO, attenuates ATM-dependent formation of γH2AX and the phosphorylation of KAP1 selectively in β-cells (Fig. 1, *A* and *B*). Like ATM-dependent signaling, NO attenuates hydroxyurea (ribonucleotide reductase inhibitor)-stimulated checkpoint kinase (Chk)1 phosphorylation in INS 832/13 cells but not MEF (Fig. 1, *C* and *D*) (12).

NO is an effective inhibitor of mitochondrial respiration (aconitase and complex IV of the ETC) (14, 15), and consistent with mitochondrial metabolism as a target for the inhibition of DDR signaling by NO, complex I inhibitor rotenone attenuates ATM- and ATR-dependent DDR signaling only in INS 832/13 cells (Fig. 1, *A* and *C*) (12, 16). We have shown that the inhibitory actions of NO and rotenone on DDR signaling are selective for β-cells (10, 12, 16), and consistent with these previous findings, inhibitors of mitochondrial respiration do not modify ATM or ATR signaling in MEF (Fig. 1, *B* and *D*). In fact, NO activates ATM and ATR signaling in non–β-cells, whereas it fails to stimulate γH2AX formation or KAP1 phosphorylation in INS 832/13 cells (Fig. 1, *A*–*D*) (10, 12). Importantly, these cell type–selective responses have been observed in rat islet cells (Fig. 1*E*) (23). Using hydrogen peroxide to induce DNA-strand breaks and DDR activation (note that camptothecin does not activate DDR signaling in primary β-cells as they are terminally differentiated and do not readily divide), γH2AX formation is observed in both insulin-containing and non–insulin-containing cells (Fig. 1*E*). DPTA/NO and rotenone attenuate hydrogen peroxide–induced γH2AX formation in insulin-containing primary β-cells but do not inhibit γH2AX formation in non–insulin-containing islet cells (Fig. 1*E*). These findings show that inhibitors of mitochondrial respiration (NO and rotenone) attenuate DDR signaling in a β-cell–selective manner in both insulinoma cells and primary β-cells (10, 12, 16, 23).

**Figure 2:**
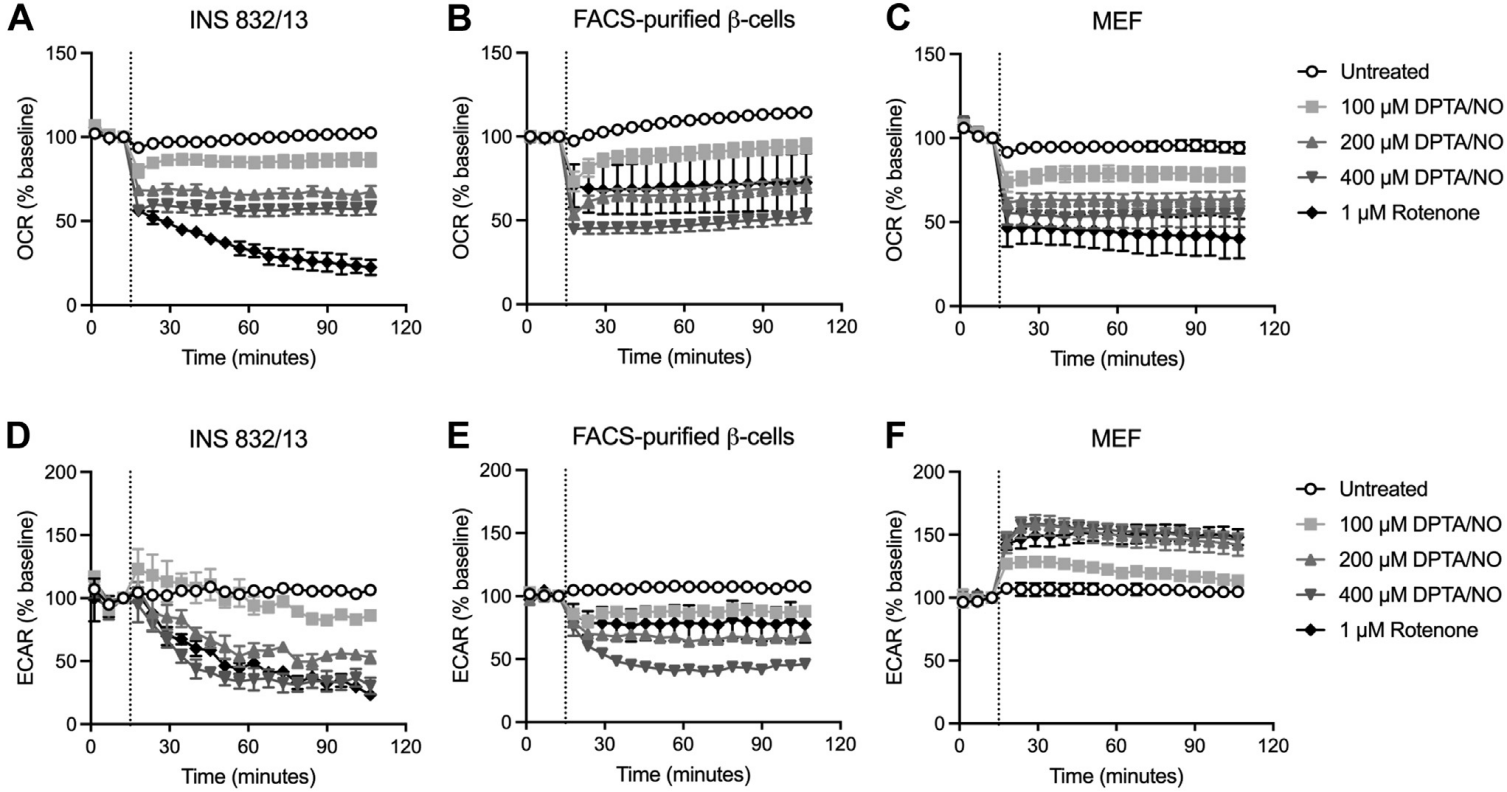
**Cell type–selective effects of nitric oxide (NO) and rotenone on glycolytic flux and mitochondrial respiration.** The oxygen consumption rate (OCR) and extracellular acidification rate (ECAR) of INS 832/13 cells (*A* and *D*), FACS-purified rat β-cells (*B* and *E*), and MEF (*C* and *F*) were measured over a 105 min incubation by extracellular flux analysis. DPTA/NO (100–400 μM) or rotenone (1 μM) was added following a 15 min incubation as indicated by the *vertical dotted line*. Results are the average ± SD of three independent experiments. DPTA, (Z)-1-[*N*-(3-aminopropyl)-*N*-(3-ammoniopropyl)amino]diazen-1-ium-1,2-diolate; FACS, fluorescence-activated cell sorting; MEF, mouse embryonic fibroblasts.

Unlike most cell types, pancreatic β-cells lack the ability to compensate for impaired mitochondrial respiration with an increase in glycolytic metabolism (12, 16). Consistent with this view, NO decreases the oxygen consumption rate (OCR) of INS 832/13 cells, fluorescence-activated cell sorting (FACS)–purified primary rat β-cells, and non–β-cells (MEF) in a concentration-dependent manner (Fig. 2, *A*–*C*). However, MEF compensate for this inhibition of mitochondrial respiration with an increase in extracellular acidification rate (ECAR; an index of glycolytic flux; Fig. 2*F*). INS 832/13 cells and FACS-purified rat β-cells lack this metabolic flexibility and do not increase ECAR (Fig. 2, *D* and *E*). In fact, DPTA/NO, at concentrations above 200 μM, decreases ECAR in INS 832/13 cells and FACS-purified β-cells. Like the actions of NO, rotenone also decreases OCR in both β-cells and non–β-cells, yet only non–β-cells (MEF) maintain the metabolic flexibility to increase glycolytic flux as assessed by ECAR (Fig. 2*F*). Using both insulinoma cells and primary rat β-cells, these findings correlate the inhibition of DDR signaling with an inability of β-cells to increase glycolytic flux in the presence of inhibitors of mitochondrial oxidative metabolism.

### Effects of inhibition of mitochondrial respiration on ATP, NAD^+^, and NADH

**Figure 3:**
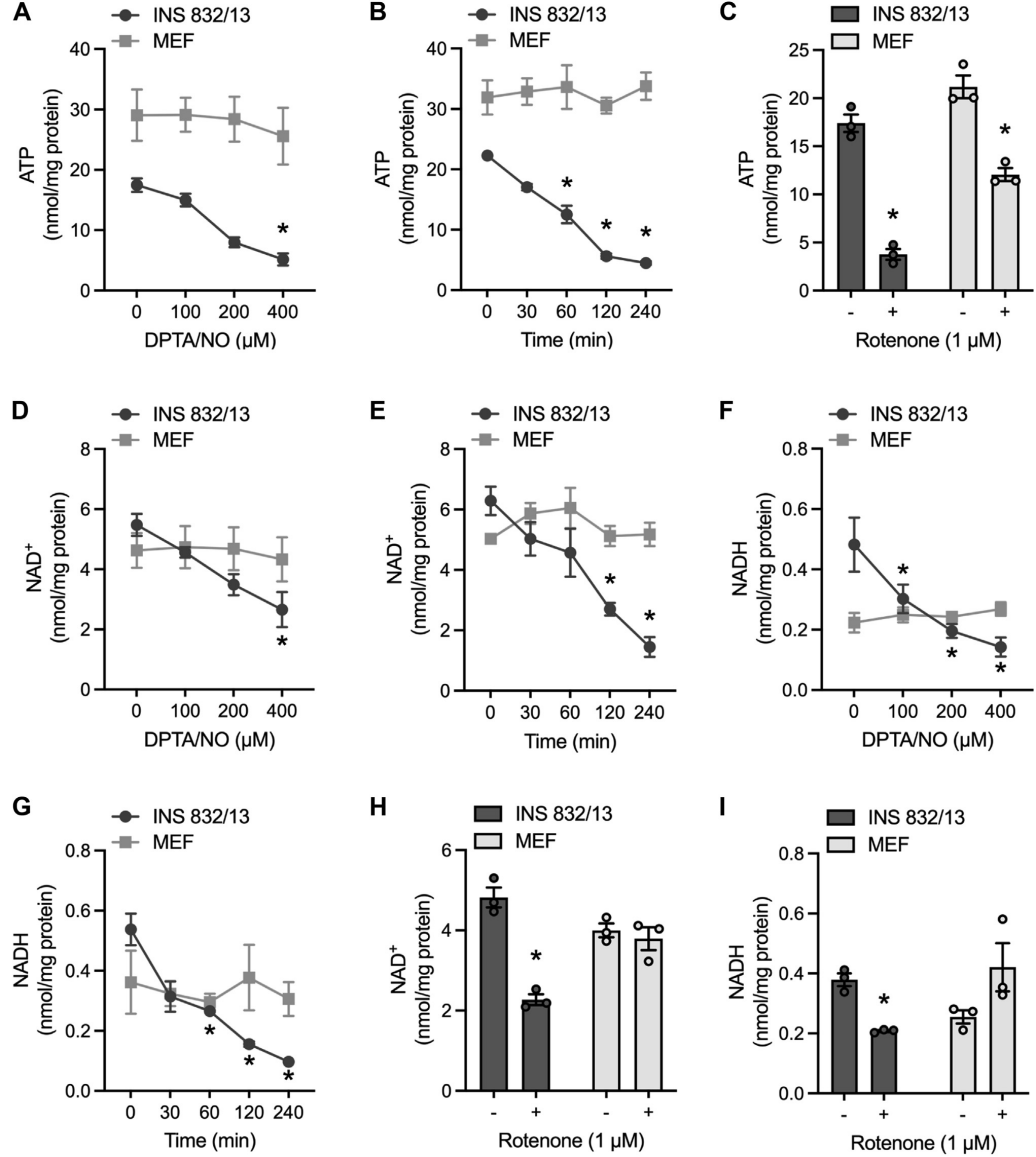
**Effects of nitric oxide (NO) and rotenone on ATP, NAD**^**+**^**, and NADH levels in INS 832/13 cells and mouse embryonic fibroblasts(MEF).** INS 832/13 cells and MEF were treated with DPTA/NO or rotenone at the indicated concentrations for 2 h (*A*, *C*, *D*, *F*, *H*, and *I*) or were treated with 400 μM DPTA/NO for the indicated times (*B*, *E*, and *G*). ATP (*A*–*C*), NAD^+^ (*D*, *E*, and *H*), and NADH (*F*, *G*, and *I*) were determined by HPLC and normalized to total protein. Results are the average ± SD of three independent experiments. Statistically significant decreases in nucleotides by DPTA/NO or rotenone compared with the untreated condition in each cell type are indicated (∗*p* < 0.05). DPTA, (Z)-1-[*N*-(3-aminopropyl)-*N*-(3-ammoniopropyl)amino]diazen-1-ium-1,2-diolate.

In most cell types, glycolytic metabolism is increased when mitochondrial oxidation is impaired (17, 18); however, the coupling of these pathways in β-cells allows for the rates of mitochondrial oxidation of glucose to determine the amount of insulin to be released for the clearance of blood glucose (19, 20, 21). One consequence of this coupling is that the inhibition of mitochondrial oxidation causes a decrease in the levels of ATP in β-cells (12, 16, 24). As shown in Figure 3*A*, DPTA/NO decreases ATP levels in INS 832/13 cells in a concentration-dependent manner that correlates with the concentration-dependent inhibition of OCR (Fig. 2*A*). In response to 400 μM DPTA/NO, there is a time-dependent loss of ATP that is first observed following a 30 min incubation and maximal following a 120 min incubation of INS 832/13 cells (Fig. 3*B*). ATP levels are maintained in MEF treated with increasing concentrations of DPTA/NO (Fig. 3, *A* and *B*), consistent with an increase in glycolytic metabolism when mitochondrial respiration is impaired (Fig. 2*F*). Like NO, rotenone also decreases ATP levels over three fold in INS 832/13 cells, whereas it is less effective at decreasing the levels of ATP in MEF (Fig. 3*C*) (12, 16).

The switch to glycolytic metabolism when oxygen is limiting or when mitochondrial oxidative metabolism is impaired requires the generation of NAD^+^ by lactate dehydrogenase (LDH) for continued activity of the NAD^+^-dependent enzyme GAPDH (17). β-cells express low levels of LDH and the monocarboxylic acid transporter (MCT), which transports pyruvate and lactate (20, 25), and lack the capacity to regenerate NAD^+^ under conditions of impaired mitochondrial function or limitations in the amount of oxygen (12, 16). In fact, LDH and MCT are considered disallowed genes in healthy adult β-cells (26). Consistent with this regulation of metabolism, NO decreases NAD^+^ levels in a concentration- and time-dependent manner in INS 832/13 cells but does not decrease NAD^+^ levels in MEF (Fig. 3, *D* and *E*). NO also decreases NADH levels in INS 832/13 cells but not MEF (Fig. 3, *F* and *G*). Like NO, rotenone also decreases NAD^+^ and NADH in INS 832/13 cells without modifying the levels in MEF (Fig. 3, *H* and *I*). These findings correlate the β-cell–selective inhibition of DDR signaling by NO and rotenone with a β-cell–selective decrease in ATP, NAD^+^, and NADH.

### The effects of NO and rotenone on the steady-state levels of glycolytic, TCA cycle, and PPP intermediates

**Figure 4:**
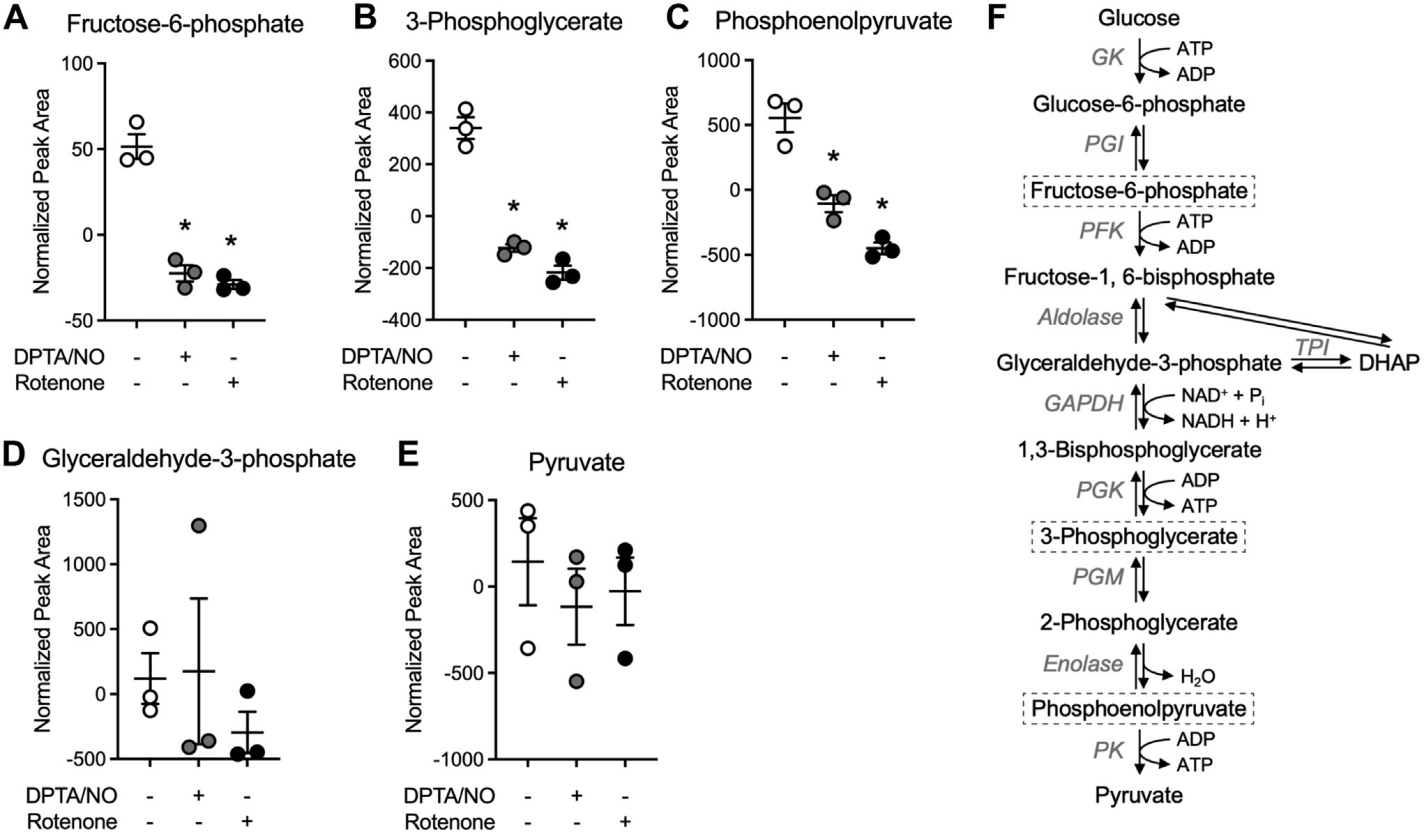
**Effect of nitric oxide (NO) and rotenone on the steady-state levels of glycolytic metabolites in INS 832/13 cells.** INS 832/13 cells (*A*–*E*) were treated with 400 μM DPTA/NO or 1 μM rotenone for 2 h. Samples were collected, and glycolytic metabolite levels were determined by mass spectroscopic analysis. Metabolites that were significantly different in response to both DPTA/NO and rotenone are boxed in the glycolytic pathway (*F*). Results are the average ± SD of three independent experiments. Statistically significant decreases in metabolites by DPTA/NO or rotenone compared with the untreated control condition are indicated (∗*p* < 0.05). DPTA, (Z)-1-[*N*-(3-aminopropyl)-*N*-(3-ammoniopropyl)amino]diazen-1-ium-1,2-diolate.

**Figure 5:**
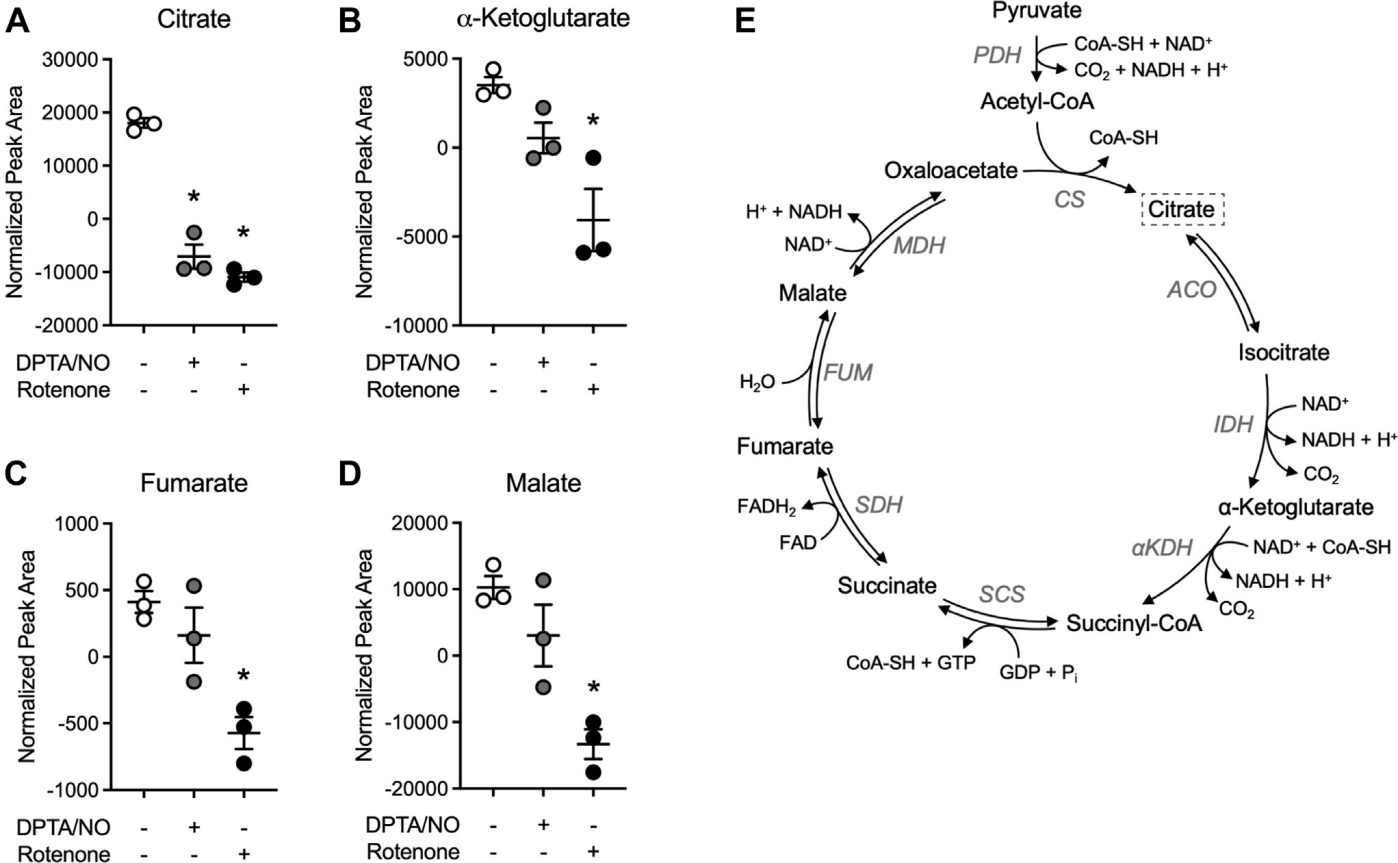
**Effect of nitric oxide (NO) and rotenone on the steady-state levels of tricarboxylic acid (TCA) cycle intermediates in INS 832/13 cells.** INS 832/13 cells (*A*–*D*) were treated with 400 μM DPTA/NO or 1 μM rotenone for 2 h. Metabolites were collected, and TCA cycle metabolite levels were determined by mass spectroscopic analysis. Citrate was the only metabolite that changed in the same manner by both DPTA/NO and rotenone and is boxed in the schematic of the TCA cycle (*E*). Results are the average ± SD of three independent experiments. Statistically significant decreases in metabolites by DPTA/NO or rotenone compared with the untreated condition are indicated (∗*p* < 0.05). DPTA, (Z)-1-[*N*-(3-aminopropyl)-*N*-(3-ammoniopropyl)amino]diazen-1-ium-1,2-diolate.

Targeted metabolomics was performed to identify changes in the levels of metabolites that may contribute to the NO-mediated cell type–selective impairment in DDR signaling. Specifically, our goal was to identify changes in metabolite levels that occur in a similar manner following treatment with NO and rotenone, as both mitochondrial inhibitors attenuate DDR signaling selectively in β-cells. Consistent with an impairment in glycolytic metabolism as evidenced by decreases in both ATP and NAD^+^ (Fig. 3), both mitochondrial toxins decrease the levels of glycolytic intermediates fructose-6-phosphate, 3-phosphoglycerate, and phosphoenolpyruvate in INS 832/13 cells (Fig. 4, *A*–*C*). The levels of the remaining intermediates of glycolysis were either below the limits of detection of the assay or not significantly changed following treatment with NO and rotenone (Fig. 4, *D* and *E*). When examining mitochondrial intermediates, only citrate levels are decreased by both NO and rotenone (Fig. 5*A*). Citrate synthase catalyzes the irreversible reaction of oxaloacetate with acetyl-CoA (produced in glycolysis) to produce citrate, suggesting that the loss of this intermediate is possibly because of the inhibition of a pathway providing substrate to the TCA cycle, specifically the oxidation of pyruvate to acetyl-CoA by pyruvate dehydrogenase complex. Rotenone causes a statistically significant decrease in the TCA cycle intermediates α-ketoglutarate, fumarate, and malate, and there is a small decrease in these metabolites in response to NO that did not achieve statistical significance (Fig. 5, *B*–*D*). NO inhibits aconitase and complex IV of the ETC, whereas rotenone inhibits complex I of the ETC but does not inhibit the TCA cycle, suggesting that carbons derived from glucose entering the TCA cycle can be oxidized in the presence of rotenone; however, this oxidation is impaired by NO because of aconitase inhibition. Overall, these findings correlate the inhibition of DDR signaling by NO and rotenone with decreases in ATP, NAD^+^, NADH, glycolytic intermediates, and citrate.

**Figure 6:**
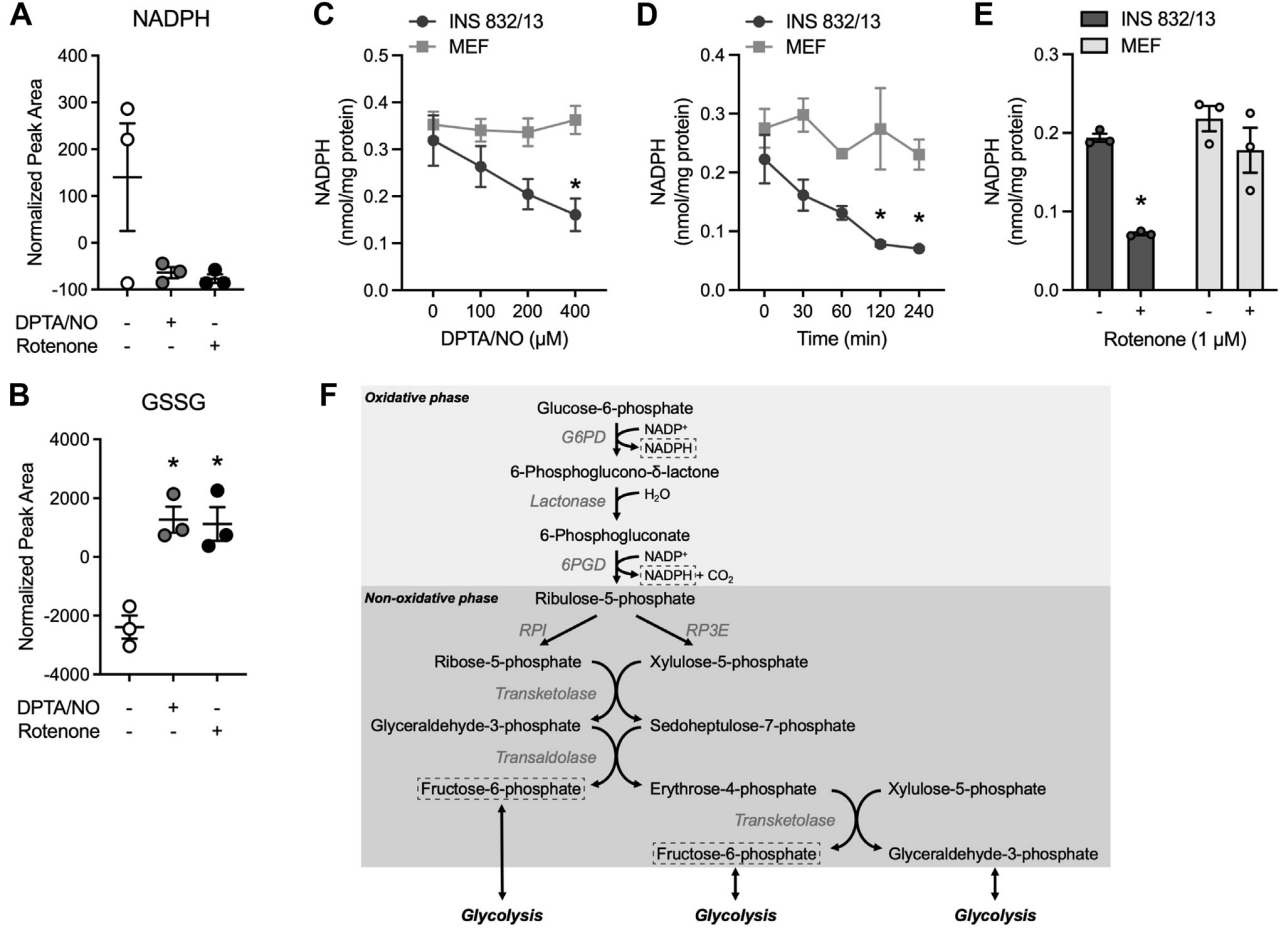
**Mitochondrial inhibitors decrease NADPH levels in INS 832/13 cells but not MEF.***A* and *B*, INS 832/13 cells were treated with 400 μM DPTA/NO or 1 μM rotenone for 2 h. Samples were collected, and changes in NADPH and GSSG levels were determined by mass spectroscopic analysis. *C*–*E*, INS 832/13 cells and MEF were treated with DPTA/NO or rotenone at the indicated concentrations for 2 h (*C* and *E*) or with 400 μM DPTA/NO for the indicated times (*D*). NADPH was determined *via* HPLC and normalized to total proteins. *F*, A schematic diagram of pentose phosphate pathway (PPP) (oxidative and nonoxidative phases) is shown with metabolites of the pathway that are decreased by NO and rotenone highlighted in *dashed boxes*. Results are the average ± SD of three independent experiments. Statistically significant decreases in metabolites by DPTA/NO or rotenone compared with the untreated condition in each cell type are indicated (∗*p* < 0.05). DPTA, (Z)-1-[*N*-(3-aminopropyl)-*N*-(3-ammoniopropyl)amino]diazen-1-ium-1,2-diolate; MEF, mouse embryonic fibroblasts.

The oxidative branch of the PPP produces NADPH (for antioxidant defense), and the nonoxidative branch provides phosphorylated intermediates that can be used to support several metabolic pathways including intermediary metabolism. The decreases in glycolytic intermediates fructose-6-phosphate, 3-phosphoglycerate, and phosphoenolpyruvate coupled with decreases in ATP and NAD^+^ led us to hypothesize that the PPP activity may be inhibited by NO and rotenone in a β-cell–selective manner. Consistent with this hypothesis, NADPH levels are decreased, whereas GSSG levels are increased in INS 832/13 cells treated with NO and rotenone (Fig. 6, *A* and *B*). Because we failed to detect NADPH in one of the INS 832/13 cell control metabolomic samples (Fig. 6*A*), HPLC analysis was used to confirm these findings. NO decreases NADPH levels in a concentration- and time-dependent manner, and rotenone also decreases NADPH levels in INS 832/13 cells (Fig. 6, *C*–*E*). These effects appear to be selective for β-cells as NO and rotenone do not decrease NADPH levels in MEF as measured by HPLC (Fig. 6, *C*–*E*). These findings correlate the β-cell–selective inhibition of DDR signaling by inhibitors of mitochondrial oxidative metabolism with decreases in ATP, NAD^+^, NADH, NADPH, glycolytic, TCA cycle, and PPP intermediates (see *schematic*, Figs. 4*F*, 5*E*, and 6*F*; *boxes* are intermediates decreased by NO and rotenone).

### Effects of inhibitors of mitochondrial respiration on glucose uptake

**Figure 7:**
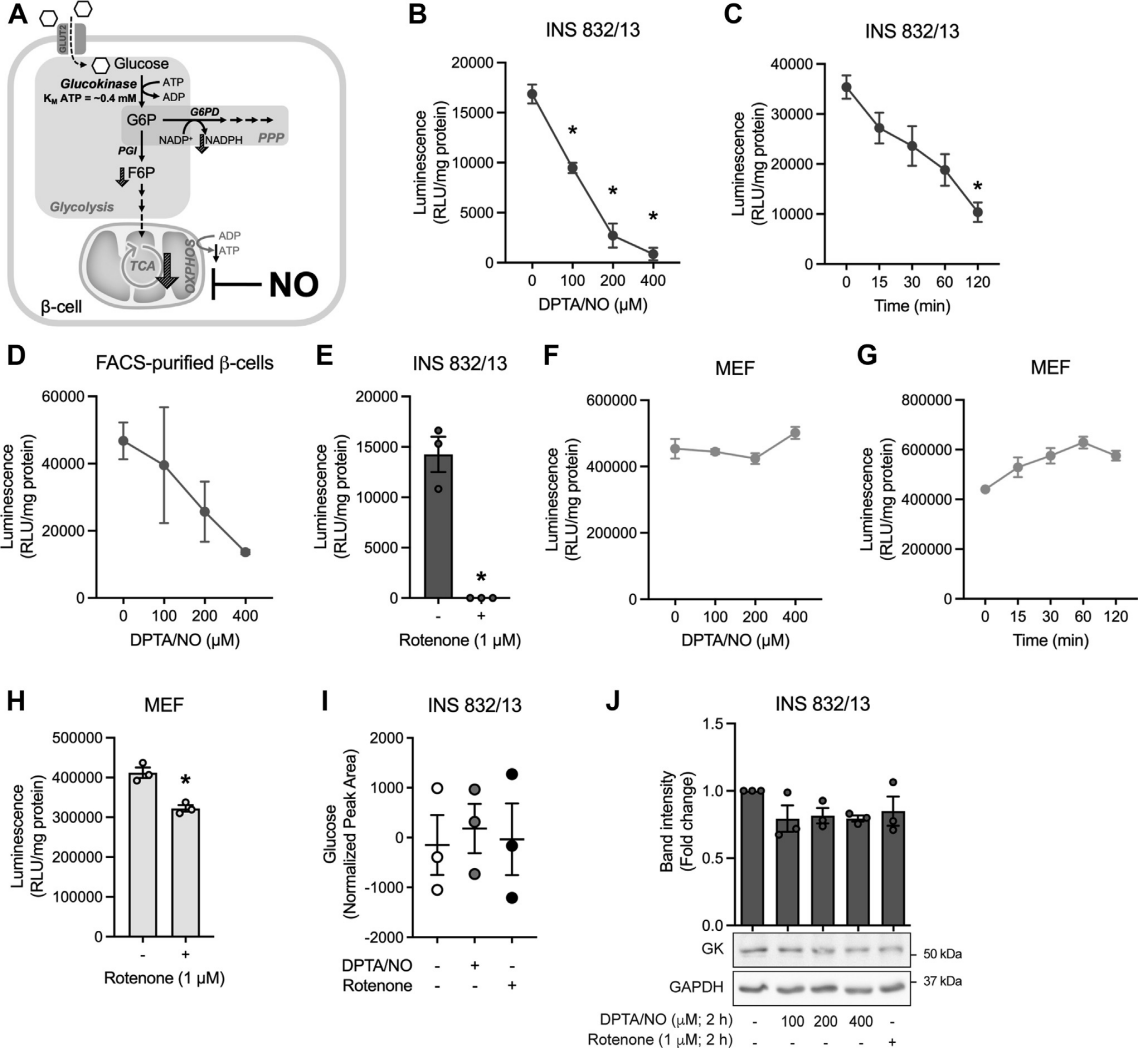
**Inhibitors of mitochondrial respiration attenuate glucose uptake selectively in β-cells.***A*, A schematic diagram representing the sites and pathways with decreased levels of metabolites in β-cells in response to nitric oxide (NO). Glucose-6-phosphate is the product of glucokinase and resides at a branchpoint between two pathways in which intermediates are decreased in response to NO and rotenone treatment (glycolysis and pentose phosphate pathway (PPP)). *B*–*H*, INS 832/13 cells (*B*, *C*, and *E*), FACS-purified rat β-cells (*D*), and MEF (*F*–*H*) were treated with DPTA/NO or rotenone at the indicated concentrations for 2 h (*B*, *D*, *E*, *F*, and *H*) or with 400 μM DPTA/NO at the indicated times (*C* and *G*). Glucose uptake was measured and normalized to total protein. *I*, Changes in INS 832/13 cell glucose levels in response to treatment with 400 μM DPTA/NO or 1 μM rotenone (2 h) were determined by mass spectroscopic analysis. *J*, INS 832/13 cell glucokinase levels are not modified following a 2 h treatment with DPTA/NO or rotenone as determined by Western blot analysis and quantified by densitometry (levels detected in untreated cells were normalized to 1). GAPDH is shown as a loading control. Results are representative (*J*) or the average ± SD (*B*–*I*, and *J* [*top*]) of three independent experiments. Statistically significant decreases in glucose uptake compared with the untreated condition in each cell type (*B*–*H*) are indicated (∗*p* < 0.05). DPTA, (Z)-1-[*N*-(3-aminopropyl)-*N*-(3-ammoniopropyl)amino]diazen-1-ium-1,2-diolate; FACS, fluorescence-activated cell sorting; MEF, mouse embryonic fibroblasts.

Glucose is transported across the plasma membrane by facilitated diffusion through specific glucose transporters. Once transported, glucose is trapped within cells by phosphorylation catalyzed by hexokinase (*K*_*M*_ for glucose of 0.003–0.3 mM; isoform dependent) (27) in most cell types and glucokinase (*K*_*M*_ for glucose of ∼8 mM) (28) in β-cells and hepatocytes. Because glucose-6-phosphate resides at the branchpoint between glycolysis and the PPP (Fig. 7*A*) and intermediates of both pathways are decreased, the effects of inhibitors of mitochondrial respiration on glucose uptake (glucose transport and phosphorylation) in β-cells and non–β-cells were examined. Glucose uptake was determined by measuring the accumulation of 2-deoxyglucose-6-phosphate, which cannot be further metabolized in cells (29). In a concentration- and time-dependent manner, NO decreases glucose uptake in INS 832/13 cells (Fig. 7, *B* and *C*) and in FACS-purified primary rat β-cells (Fig. 7*D*). Like NO, rotenone also inhibits glucose uptake in INS 832/13 cells (Fig. 7*E*). The inhibitory actions of NO on glucose uptake appear to be selective for β-cells, as this free radical does not inhibit glucose uptake in MEF (Fig. 7, *F* and *G*), and rotenone has only a minor inhibitory effect on glucose uptake in MEF (∼25% decrease, Fig. 7*E* *versus* Fig. 7*H*). Importantly, glucose transport into INS 832/13 cells is not modified by NO or rotenone (Fig. 7*I*), indicating that effects of NO on metabolism are due to a decrease in glucose uptake (phosphorylation by glucokinase) in β-cells. In further support of this conclusion, the steady-state levels of glucokinase are not changed by treatment with NO or rotenone (Fig. 7*J*). Together, these findings suggest that NO limits glucokinase activity and decreases glucose phosphorylation, leading to shutdown of glucose metabolism that is associated with the β-cell–selective inhibition of DDR signaling by NO.

## Discussion

Pancreatic β-cells are found in the highly vascularized islets of Langerhans. They are responsible for the sensing of blood glucose levels and secreting the appropriate amount of insulin to facilitate the clearance of glucose from the blood stream. Glucose is sensed through the activity of the low-affinity high-turnover Glut2 transporter that allows for glucose entry into β-cells in a concentration-dependent manner, and glucokinase (high *K*_*M*_ for glucose), which phosphorylates glucose, trapping it in β-cells (30). In addition to this sensing blood glucose levels, the amount of insulin that is secreted is controlled by the rates of glucose oxidation (20, 21). Nearly all the carbons of glucose are oxidized completely to CO_2_ in β-cells, and the rates of oxidation increase with increases in glucose levels (19, 20, 21). Most cell types only utilize mitochondrial oxidative metabolism when there is a demand for ATP or intermediates produced by anaplerotic reactions in the mitochondria (17). The coupling of glycolysis to mitochondrial oxidative metabolism in β-cells is facilitated by the relative absence of LDH (20) and the inability to regenerate NAD^+^ for continued glycolysis (GAPDH reaction) when oxygen is limiting or mitochondrial oxidative metabolism is impaired (12, 16). This metabolic coupling serves as an important physiological regulator that prevents inappropriate insulin production under conditions where hepatic glucose production is necessary (31). In this report, we identify a second potential physiological role by which the regulation of intermediary metabolism controls β-cell responses to external stimuli. In this case, NO limits DDR signaling and DDR-dependent β-cell apoptosis by inhibiting mitochondrial oxidative metabolism (10, 12, 16).

The activation of DDR signaling in β-cells in response to DNA damage leads to apoptosis (10), and we have shown that NO, when produced at micromolar levels or iNOS-derived levels, inhibits DDR signaling (Fig. 1, *A* and *C*) and prevents DDR-induced apoptosis in a β-cell–selective manner (10, 12, 16). This action of NO is selective for β-cells and has been correlated with the inhibition of mitochondrial oxidative metabolism and loss of ATP (10, 12, 16). In support of this conclusion, we have shown that inhibitors of mitochondrial respiration (rotenone, oligomycin, antimycin A, and FCCP) attenuate DDR signaling selectively in β-cells (Fig. 1, *A* and *C*) (12, 16). As described previously, there is a coupling of glycolysis and mitochondrial oxidative metabolism such that most of the carbons of glucose are oxidized to CO_2_ and the rates of oxidation increase with increasing concentrations of glucose (19, 20, 21). This allows the β-cell to couple the rate of insulin secretion to the rate of mitochondrial glucose oxidation (19, 20, 21). Because of this coupling, β-cells lack the ability to compensate for impaired mitochondrial respiration by increasing glycolytic flux (Fig. 2, *D* and *E*), resulting in a decrease in ATP (Fig. 3, *A*–*C*) (12, 16). Non–β-cells lack this coupling and have the metabolic flexibility to increase glycolytic flux when mitochondrial oxidative metabolism is impaired (Fig. 2*F*) and by this mechanism maintain ATP levels (Fig. 3, *A*–*C*) (12, 16).

It is well known that β-cells express LDH at low levels, and they do not express the lactate/pyruvate transporter MCT (20, 25, 26). As expected, NAD^+^ levels fall in β-cells treated with NO and mitochondrial respiratory inhibitors (Fig. 3, *D* and *E*). In addition to LDH, the cytosolic pool of NAD^+^ is generated through the glycerol-3-phophate shuttle and malate–aspartate shuttle, whereas the mitochondrial pool of NAD^+^ can be generated by the ETC. Under impaired mitochondrial oxidative metabolism, NAD^+^ cannot be generated through glycerol-3-phosphate shuttle or malate–aspartate shuttle, as both pathways are linked to mitochondrial oxidative metabolism. Consequently, the inhibition of mitochondrial respiration shuts down pathways that generate NAD^+^ in β-cells. Because NAD^+^ is a required cofactor of the glycolytic enzyme GAPDH (see *schematic*, Fig. 4*F*) (18), the decreased NAD^+^ in response to NO contributes to the inability to sustain glycolysis in β-cells when mitochondrial respiration is impaired (Fig. 2, *D* and *E*) (12, 16).

It is surprising that the decrease in NAD^+^ does not correlate with an increase in NADH. In fact, we show that NADH levels are also decreased in β-cells treated with NO and inhibitors of mitochondrial respiration (Fig. 3, F, G, and I). NADH is generated in glycolysis and the TCA cycle through the reduction of NAD^+^, and the shutdown of glycolysis and the TCA cycle may explain the decreased NADH levels in β-cells. Furthermore, these results suggest that the decrease in NAD^+^ and NADH is not simply associated with the redox state of the nucleotide pools. Enzymes such as sirtuins (Sirts), poly(ADP-ribose) polymerase (PARP), and CD38 utilize NAD^+^ without oxidizing it back to NADH; instead, nicotinamide is generated (32). It is possible that the decreases in NAD^+^ and NADH in β-cells are due to an increase in the activity of these enzymes, resulting in the depletion of the NAD^+^ pool, whereas the inhibition of mitochondrial oxidative metabolism attenuates NADH regeneration. NO has been shown to activate PARP in many cell types, including β-cells; however, it has been our experience that PARP does not contribute to the actions of cytokine or cytokine-derived NO on β-cell viability (33).

Targeted metabolomic analysis in INS 832/13 cells exposed to NO or rotenone was employed to identify metabolites that are changed in a similar manner in β-cells. NO and rotenone decrease glycolytic (Fig. 4), TCA cycle (Fig. 5), and PPP (Fig. 6) intermediates in INS 832/13 cells. Glucose-6-phosphate is found at a metabolic branchpoint in glucose metabolism as it is a substrate for glycolysis and the PPP. Decreases in glycolytic intermediates and NADPH (PPP) suggest that NO and rotenone inhibit glucose metabolism upstream of glucose-6-phosphate. This hypothesis is also consistent with decreases in NAD^+^ and NADH. Glucose is transported into cells by facilitated diffusion *via* GLUT transporters (Glut2 in β-cells), and this process is not inhibited by NO or rotenone in β-cells (Fig. 7*I*); however, glucose uptake is inhibited (Fig. 7, *B*–*E*). The first step in the metabolism of glucose is phosphorylation by hexokinase (four different isoforms I–IV), trapping glucose in cells (22). β-cells express glucokinase (hexokinase IV), which has *K*_*M*_ for glucose of ∼8 mM and ATP ∼0.4 mM (28). Under normal conditions, basal ATP levels in β-cells are ∼1 to 2 mM (34, 35) or well above the *K*_*M*_ for ATP of glucokinase, leaving glucose as the limiting factor regulating glucokinase activity (28). However, under conditions where mitochondrial respiration is inhibited (by NO or respiratory chain inhibitors), ATP levels decrease 8-fold to 10-fold or to levels (∼0.1–0.2 mM) well below the *K*_*M*_ of glucokinase for ATP. This results in a decrease in the activity of glucokinase and a decrease in glucose uptake. In support of this conclusion, we showed that NO and rotenone decrease glucose uptake selectively in INS 832/13 cells and primary rat β-cells purified by FACS (Fig. 7, *B*–*H*) without reducing glucokinase expression (Fig. 7*J*). Like glucokinase, hexokinase I, II, and III have a high *K*_*M*_ for ATP of 0.5 to 1.0 mM (27) and should also be sensitive to inhibition in the absence of sufficient ATP levels; however, non–β-cells possess the metabolic flexibility to enhance glycolytic flux in the presence of mitochondrial inhibitors (Fig. 2*F*) and maintain ATP to levels sufficient to support continued glycolysis (Fig. 3, *A*–*C*) (12, 16). In further support of this hypothesis, we have shown that when MEF are forced to generate ATP *via* mitochondrial oxidative metabolism of glutamine (culturing in glucose-free galactose-containing medium), inhibitors of mitochondrial respiration (NO and rotenone) attenuate DDR signaling (ATM and ATR), and this inhibition correlates with a loss of ATP (12, 16). Also, NO no longer activates DDR signaling or stimulates apoptosis in galactose-cultured MEF (12, 16).

The mechanisms by which decreased levels of ATP and glucose uptake attenuate DDR signaling are unknown. While protein phosphatase 1 is a regulator of DDR signaling, we have shown that it does not contribute to the inhibitory actions of NO on ATM signaling (23). Furthermore, we do not believe that it is simply a decrease in ATP to levels below the *K*_*M*_ of DDR kinases (*e.g.*, *K*_*M*_ of ATM for ATP ∼25 μM), as the levels of this nucleotide are more than sufficient to support DDR signaling (36). NO at concentrations that limit DDR signaling and DDR-directed apoptosis also stimulates growth arrest and DNA damage–inducible protein (GADD)45α-dependent DNA repair in β-cells (37). GADD45α expression and GADD45α-dependent repair of damaged β-cell DNA in response to NO is Forkhead Box O1 (FoxO1)- and Sirt1-dependent (38). Under normal conditions, FoxO1 is phosphorylated by PI3K/Akt signaling and is sequestered in the cytoplasm by the scaffolding protein 14-3-3 (39). When dephosphorylated, FoxO1 is released and translocates to the nucleus to activate gene expression (39). NO is an inhibitor of PI3K signaling at low micromolar levels, allowing for FoxO1 dephosphorylation and nuclear translocation (38). Furthermore, increases in Sirt1 activity appear to be required for NO-stimulated FoxO1-dependent GADD45α expression (38). We hypothesize that NO functions to inhibit DDR signaling while activating base excision repair of damaged DNA in β-cells (37). Additional studies directed at the regulation of Sirt1, FoxO1, and DDR signaling will be required to fully understand the interplay between these pathways and the cell type specificity of this regulation.

NO appears to place β-cells in a state of “suspended animation,” or a condition in which glucose uptake (Fig. 7), oxidative metabolism, and glucose-stimulated insulin secretion are impaired, but β-cells remain viable. The concentrations of NO that are required to induce this state are levels known to be produced by β-cells following iNOS induction and fall in the high nanomolar to low micromolar levels (0.8–5 μM). These physiological concentrations of NO are also generated using DPTA/NO at 200 to 400 μM (10). While β-cells become metabolically impaired under these conditions, they do not die. Removal of NO by washing or the addition of NO synthase inhibitors to islets pretreated for 18 to 24 h with cytokines results in the complete recovery of mitochondrial oxidative metabolism and insulin secretory function and the repair of damaged DNA (40, 41, 42). NO also activates heat shock and unfolded protein responses in β-cells (42, 43), and one consequence of the activation of these stress responses is the impairment in cytokine signaling. We have shown that heat shock, NO, and endoplasmic reticulum stress inducers impair the activation of nuclear factor κB in response to IL-1 and the phosphorylation of signal transducers and activators of transcription 1 in response to interferon-γ (42, 43, 44). In the context of “suspended animation” induced by iNOS-derived levels of NO, intermediary metabolism is impaired, stress responses are stimulated, and β-cells become refractory to proinflammatory cytokines and resistant to apoptosis.

In addition to the aforementioned roles in the inhibition of apoptosis and cytokine singling, NO also limits picornavirus replication in a β-cell–selective manner (45, 46). The inhibition of virus replication requires iNOS-derived levels of NO and is associated with an inhibition of mitochondrial oxidative metabolism and decrease in ATP (45, 46). Much like DDR signaling, multiple inhibitors of mitochondrial respiration (*e.g.*, rotenone, antimycin A, FCCP) also attenuate picornavirus replication in a β-cell–selective manner (45, 46). Members of the picornavirus family have been proposed to participate in the initiation events or triggering events that initiate autoimmune diabetes (47).

**Figure 8:**
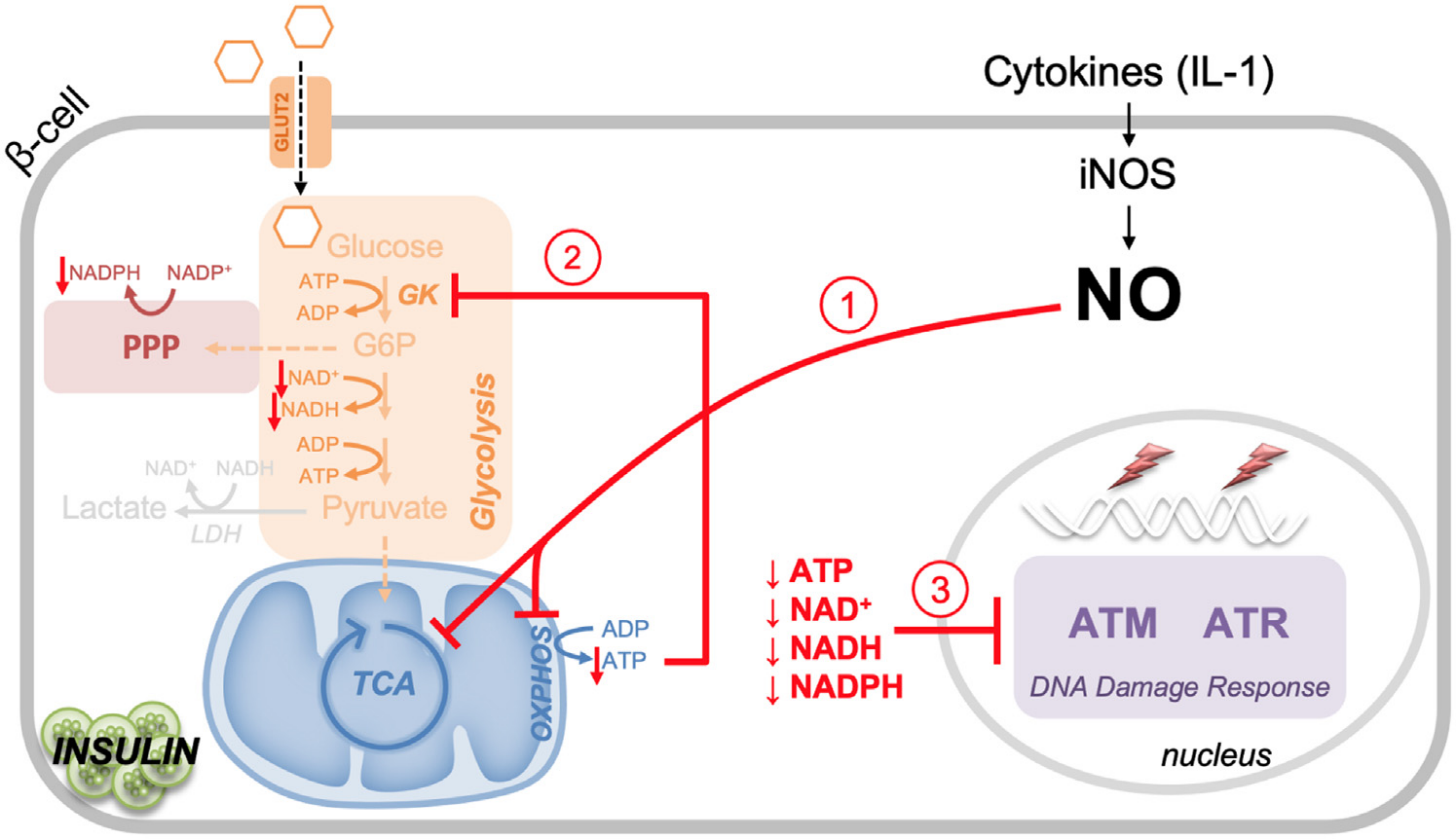
**Schematic.** Nitric oxide inhibits mitochondrial respiration through inhibiting aconitase of the tricarboxylic acid (TCA) cycle and complex IV of the electron transport chain (ETC), resulting in a loss of ATP in β-cells (action 1). Because of the high *K*_*M*_ of glucokinase for ATP (∼0.4 mM), ATP levels become limiting for glucokinase activity. This results in decreases in the rates of oxidation of glucose-6-phosphate in glycolysis and the pentose phosphate pathway (PPP) (action 2) decreasing NAD^+^, NADH, and NADPH levels in β-cells. The cell type–selective action is associated with the lack of glycolytic compensation for impaired mitochondrial oxidation in β-cells, whereas ATP, NAD^+^, NADH, and NADPH levels are maintained in non–β-cells because of glycolytic compensation for impaired mitochondrial oxidation (action 3). The net effect is a β-cell–selective metabolic regulation of DNA damage response signaling that is controlled by the actions of nitric oxide.

Overall, these studies provide additional evidence that NO, *via* the inhibition of intermediary metabolism, attenuates DDR signaling in β-cells by decreasing ATP to levels that fail to support the phosphorylation of glucose by glucokinase (Fig. 8). The loss of ATP is associated with a coupling of glycolysis and mitochondrial respiration, which is essential for β-cell function. In non–β-cells, NO does not limit DDR signaling or decrease the levels of ATP, as non–β-cells enhance glycolytic metabolism as a compensatory mechanism for impaired mitochondrial oxidative metabolism (12, 16). This cell type–selective regulation or coupling of glycolysis and mitochondrial oxidative metabolism, while essential for insulin secretion, is used by β-cells to defend against apoptosis (10) and to limit the replication of viruses from a family thought to be important in the initiation of autoimmune diabetes (48, 49). NO is produced at micromolar levels by β-cells following cytokine stimulation and iNOS induction, and many groups have reported that cytokine-induced β-cell apoptosis (50, 51) is a contributory factor in the development of autoimmune diabetes (52, 53). It is our experience, as well as that of others, that it is challenging to kill primary β-cells by apoptosis (54, 55, 56), and that NO is a potent inhibitor of apoptosis (10, 16, 43). Furthermore, recent single-cell RNA-sequencing of mouse islet cells show that cytokines fail to stimulate the expression of proapoptotic factors (57, 58). In fact, IL-1 and interferon-γ stimulate antiviral genes in all endocrine cells, not just β-cells (57, 58). Given that β-cells reside in a highly vascularized micro-organ that is essential in the regulation of blood glucose levels, and that they are exposed to cytokines during infections (*e.g.*, inflammatory viruses such as picornavirus and coronavirus), cytokine signaling in β-cells likely serves important physiological roles. Our findings suggest that these roles may include the expression of iNOS and production of micromolar levels of NO, which place β-cells in a state of “suspended animation” where cellular function and oxidative metabolism are decreased, they become resistant to cytokine signaling and are capable of limiting virus replication; however, they do not die but maintain the capacity to fully recover metabolic and secretory functions (40, 41, 59). We hypothesize that it is when the cytokine storm is persistent or the presence of genetic defects that limit the protective host responses activated by cytokines and NO that β-cells are lost. Our findings begin to identify additional roles for the regulation of intermediary metabolism, in addition to its critical role in the regulation of insulin secretion, in the protection of β-cells from damage and infection.

## Experimental procedures

### Cell lines, animals, and materials

INS 832/13 cells were obtained from Dr Christopher Newgard (Duke University). MEF were purchased from American Type Culture Collection. Male Sprague–Dawley rats were purchased from Harlan. Connaught Medical Research Laboratories (CMRL) 1066 medium and β-mercaptoethanol were purchased from Thermo Fisher Scientific. RPMI1640 medium, Dulbecco's modified Eagle's medium, trypsin (0.05% in 0.53 mM EDTA), l-glutamine, sodium pyruvate, Hepes, and penicillin–streptomycin were purchased from Corning. Fetal bovine serum was purchased from HyClone. DPTA/NO was purchased from Cayman Chemical. 2-Deoxyglucose (2-DG), camptothecin, hydroxyurea, and rotenone were purchased from MilliporeSigma. Primary and secondary antibodies used for Western blot and immunofluorescence were purchased as follows: mouse anti-γH2AX (Ser139) from MilliporeSigma; rabbit anti-phospho-KAP1 (Ser824) and rabbit anti-glucokinase from Abcam; rabbit anti-phospho-Chk1 (Ser345) from Cell Signaling Technology; mouse anti-GAPDH from Thermo Fisher Scientific; guinea pig anti-insulin from DakoCytomation; horseradish peroxidase (HRP)–conjugated donkey anti-mouse, HRP-conjugated donkey anti-rabbit, and Cy3-conjugated donkey anti-mouse from Jackson ImmunoResearch Laboratories, Inc; and, Alexa Fluor 488–conjugated donkey anti-guinea pig from Molecular Probes.

### Culture of cell lines and primary islet cells

INS 832/13 cells and MEF were cultured as previously described (60). Islets were isolated from male Sprague–Dawley rats by collagenase digestion as previously described (61, 62) and cultured overnight in complete CMRL (CMRL-1066 containing 10% fetal bovine serum, 2 mM glutamine, 50 U/ml penicillin, and 50 μg/ml streptomycin). Islets were dispersed into individual cells by treatment with trypsin in Ca^2+^- and Mg^2+^-free Hanks (63). Dispersed islet cells were incubated for 60 min at 37 °C in complete CMRL prior to cell sorting. β-cells were isolated from rat islets by FACS as previously described (64) using a FACSMelody Cell Sorter and cultured overnight in complete CMRL prior to experimentation. Animal welfare was approved by the Institutional Animal Care and Use Committees at the Medical College of Wisconsin (A3102-01).

### Western blot analysis

Western blot analysis was performed as previously described (38), using the following antibody dilutions: 1:1000 dilution for anti-phospho-Chk1 (Ser345) and anti-glucokinase; 1:2000 dilution for anti-phospho-KAP1 (Ser824); 1:10,000 dilution for anti-γH2AX (Ser139); 1:40,000 dilution for anti-GAPDH; and 1:20,000 dilution for HRP-conjugated donkey anti-mouse and HRP-conjugated donkey anti-rabbit. Antigen was detected by chemiluminescence (65).

### Immunofluorescence

Immunofluorescence was performed as previously described (45). Images were taken using a Nikon 90i confocal microscope. Antibody dilutions were as follows: 1:500 dilution for anti-γH2AX (Ser139) and 1:1000 dilution for anti-insulin and all secondary antibodies.

### Cellular bioenergetics

OCR and ECAR were measured in FACS-purified β-cells (50,000–75,000 cells/well), INS 832/13 cells (20,000 cells/well), and MEF (10,000 cells/well) using the Seahorse XFe96 analyzer (Agilent Technologies). Measurements were made in Dulbecco's modified Eagle's medium containing 5.5 mM glucose, 2 mM pyruvate, and 1 mM glutamine and were normalized to total protein determined using the Pierce BCA protein assay kit (Thermo Scientific). Results are expressed as percent of baseline for each cell type.

### Metabolomic analysis

INS 832/13 cells were cultured for 24 h prior to treatment with the medium replaced 2 h before sample extraction. Samples were extracted in 80% methanol containing heavy labeled internal standards on dry ice–ethanol bath and were transferred to precooled 1.5 ml low-binding microfuge tubes. Extracts were incubated on dry ice–ethanol bath for 20 min and were centrifuged at 14,000*g* for 5 min at 4 °C. Supernatants were collected for analysis, whereas pellets were dissolved in 0.5 N NaOH, and total protein was quantified using the Bradford assay (Thermo Scientific).

Targeted metabolomic analysis was performed using a 1200 Infinity Series HPLC (Agilent) in-line with a 6430 QqQ (Agilent) using dynamic multiple reaction monitoring scheduling. Samples were analyzed separately for nucleotides and cofactors (assay 1) and energetic, anabolic, and catabolic intermediates (assay 2). Raw data were processed in Skyline (66). Peak areas were exported from Skyline and normalized to total protein and heavy labeled internal standards. Data were analyzed using MetaboAnalyst, no filtering was applied, and data were normalized to the control group with centering around the mean (67, 68). Statistical analysis was performed using one-way ANOVA with Fisher’s least significant difference post hoc analysis. Details of the mass spectrometry and analysis parameters are outlined in Supporting information 1.

### Nucleotide measurement

Nucleotides (ATP, NAD^+^, NADH, and NADPH) were quantified by HPLC using a SUPELCOSIL LC-18-T column (3 μm, 150 × 4.6 mm internal diameter) as previously described (69, 70, 71, 72). Nucleotide levels were normalized to total protein determined using the Pierce BCA protein assay kit and expressed in nanomoles per milligram protein.

### Glucose uptake measurement

The Glucose Uptake-Glo Assay from Promega was used to measure glucose uptake according to the manufacturer’s instructions. For these studies, INS 832/13 cells and FACS-purified rat β-cells were incubated with 15 mM 2DG for 15 min, whereas MEF were incubated with 5 mM 2DG for 10 min (incubation times and concentrations of 2DG were optimized for each cell type). Glucose uptake, expressed as relative luminescence unit, was normalized to total protein as determined using the Pierce 660 nm protein assay kit supplemented with ionic detergent compatibility reagent (Thermo Scientific).

### Statistical analysis

Statistical significance was evaluated using paired *t* test, one-way or two-way ANOVA, and Tukey’s or Sidak’s multiple comparison post hoc analysis as indicated (∗*p* < 0.05).

## Data availability

All data not included in this article will be shared upon request. Contact Dr Corbett (jcorbett@mcw.edu) for data requests.

## Supporting information

This article contains supporting information.

## Conflict of interest

The authors declare that they have no conflicts of interest with the contents of this article.

## Supporting information

Supporting information

Enzyme abbreviation
