# Supplementary material for: β-cell–selective inhibition of DNA damage response signaling by nitric oxide is associated with an attenuation in glucose uptake
Source: J Biol Chem. 2023 Feb 10;299(3):102994. doi: 10.1016/j.jbc.2023.102994 (PMC10023961; doi:10.1016/j.jbc.2023.102994)
Supplement: Supporting information [file mmc1.pdf]

Chromatography and MS Instrument Acquisition Settings	
Injection Volume	3 µL
Guard Column	SeQuant ZIC-HILIC guard column, 2.1x20mm, 5 µm particle size
Analytical Column	SeQuant ZIC-HILIC 2.1x100mm 3.5 µm particle size
Solvent A	20 mM Ammonium Acetate 0.05% Acetic Acid
Solvent B	Acetonitrile
Flow Rate	200 µL/min
Mass Spectrometer	6430 QqQ Agilent
Gas Flow	12 L/min
Nebulizer Pressure	40 psi
Positive Spray Voltage	3000 V
Negative Spray Voltage	3250 V
Gas Temperature	300 °C
Column Temperature	40 °C

Catalog #
M1504360001
M1504470001
Sigma, 73594<br>Fisher, A11350

Chromatography		
Time (min)	Solvent A	Solvent B
0	10%	90%
3.5	10%	90%
19.5	60%	40%
21.5	60%	40%
22.5	10%	90%
35	10%	90%

Assay 1

Compound Name	Precursor m/z	Product m/z	Fragmenter (V)	CE (V)	Accelerater
acetoacetyl-CoA	852	345	130	30	4
acetyl-CoA	810	303	130	27	4
ADP	426.1	159	130	21	2
AMP	348.15	136	35	12	7
ATP	505.9	159	110	30	4
C13 ALA	93	46.2	35	8	7
C13 Leu	138.1	91	35	5	7
C13 Phe	172.1	109	35	25	7
C13 Succinate	121	76	35	9	7
CMP	324	112	130	11	4
coenzyme A	768	261	130	31	2
CTP	482	384	100	15	4
FAD	786	348	75	24	2
GDP	442	79	130	22	6
GMP	362.1	79	130	16	6
GTP	522	424	75	20	2
NAD	664.1	428	125	25	4
NADH	666.1	514	130	20	6
NADP	744.2	136	120	48	4
NADPH	746.15	729	100	15	2
Nicotinamide ribotide (NMN)	335	123	100	10	4
UDP	403	79	130	21	6
UMP	325	97	35	7	6
UTP	483	159	130	23	2

Assay 2

Compound Name	Precursor m/z	Product m/z	Fragmenter (V)	CE (V)	Accelerater
2-Oxo-4-hydroxy-5-aminovalerate*	148	130	75	6	4
2-phosphoglycerate	185	79.1	75	16	2
3-(4-hydroxyphenyl)lactate	181	135	130	13	2
3-phosphoglycerate	185	97	55	14	2
4-guanidinobutanoate	146	86	100	15	4
5-Formyl-THF	474	327	100	22	2
Adenosine	268.15	136.1	100	12	6
Adenylosuccinate*	462.1	79	75	19	2
Alanine	90.1	44.2	35	8	7
alpha-hydroxyisocaproate*	131.1	85.1	100	12	2
alpha-ketogluterate	145	101	55	6	4
Aminoisobutyrate	104.1	86	75	4	4
Arginine	175.02	60	35	10	7
Argininosuccinate	291.1	70	130	18	4
Ascorbate	175	115	55	14	4
Asparagine	133.1	74	75	8	4
Aspartate	134	74	35	10	7

b-Alanine	90.25	89.95	75	1	2
Betaine	118.1	58.2	100	30	2
C13 ALA	93	46.2	35	8	7
C13 Leu	138.1	91	35	5	7
C13 Phe	172.1	109	35	25	7
C13 Succinate	121	76	35	9	7
Carbamoyl phosphate	140	79	130	8	6
Carnitine	163.1	85	100	7	4
choline	104	60	100	13	2
Citrate	191.05	87	100	17	2
citrulline	176	70	75	16	4
Creatinine	114.1	44	100	14	4
Cysteine	122.1	59.1	75	14	2
Cytosine	112.1	95	130	14	2
deoxyadenosine	252	136	100	12	4
DHAP	169	79	75	13	4
dihydroorotate	157	113	130	3	4
E4P	198.9	97.2	130	4	4
F1,6P	339	97	75	19	4
F6P	259.03	169	130	4	2
folate	442	295	130	15	2
Fructose	179	59	75	12	4
Fumarate	115	71	130	5	4
G6P	259.02	199	130	0	2
Galactose	179	59	75	12	4
Geranyl pyrophosphate	313.1	79.1	130	18	2
Glucose	179	89	35	4	4
Glucose/Galactose	179	59	75	12	4
glucose-1-phosphate	259.01	241	130	0	4
Glutamate	148.1	84.1	35	13	7
Glutamine	147.1	84.1	80	15	4
Glutathion disulfide	613	231	130	28	6
Gly	76.1	30.5	75	6	2
glyceraldehyde 3 phosphate	169.05	97	75	6	4
GSH	308.1	162	35	17	2
Guanine	152.2	110	130	15	4
Guanosine	284.1	135	75	17	6
Histidine	156.1	110.1	35	8	7
Homocysteine	136.04	90.1	75	6	2
Homoserine	120.15	44.2	75	14	2
Hydroxy proline	132.004	68.2	75	14	2
hypoxanthine	135	92	130	14	4
Imidazole	69	42.24	130	6	2
Imidazoleacetic acid	127.002	81	130	8	2
IMP	349	137	100	9	4
Inosine	267	135	130	16	4
Isocitrate	191.02	117	80	15	3
Isoleucine	132.1	86	35	5	7
Kynurenic acid	188	144	100	10	4

kynurenine	209	146	35	15	7
Lactate	89	43.2	75	10	2
Leucine	132.1	86	35	5	7
Lys	147	67	70	35	2
Malate	133	115	75	8	4
Methionine	150.1	133	35	4	7
Methyl (R)-3-methyl-2-oxopentanoate	145	57	130	15	4
Methylmalonic acid	117.1	73.1	75	8	6
methylnicotinamide	137.001	94	130	14	4
N-acetylalanine	132	44	100	14	4
N-acetyl-glutamate	190.1	84.1	55	14	4
N-acetyl-glutamine	189.1	130	100	10	4
N-acetyltryptophan	247	130	130	16	4
N-Carbamoyl-alanine	133.1	115	55	2	4
Nicotinamide	123	80	100	14	4
Nicotinate	122	78	35	8	7
O-acetyl-L-serine	148	106	55	6	2
Ornithine	133	70	75	18	4
orotate	155	111	75	9	4
Phenylalanine	166.1	103	35	25	7
Phosphoenolpyruvate	167	79	55	10	6
Proline	116.1	70.1	35	13	7
pyridoxine	170	134	100	14	6
Pyruvate	87	43	55	1	6
Quinolinate	166	122	35	6	7
R5P(ribose/ribulose)	229.1	97.05	100	5	6
riboflavin	377	243	130	20	2
S7P	288.9	97.1	100	17	2
S-adenosyl-L-homoCysteine	385.1	136	100	14	4
S-adenosyl-L-methionine	399.1	250	35	11	2
Serine	106	60	35	7	7
S-methyl-5-thioadenosine	298	136	100	13	6
Succinate	117	73	35	9	7
Threonine	120	74	35	6	7
Tryptophan	205	146	35	13	7
Tyrosine	182.1	77	75	14	4
UDP-glucose/galactose	565.1	323	130	22	2
Urea	61.1	44.2	100	12	2
Uric Acid	167.02	124	100	15	2
Uridine	243	200	130	2.5	4
Valine	118.1	55.2	35	19	7
Xanthine	153	110	75	15	4

*No commercially available standard, included in acquisition but not compounds acceptable for quantitation

Retention Time (min)	Retention Window	Polarity
15.9	6	Positive
15.7	6	Positive
19	9	Negative
16.5	7	Positive
17.5	7	Negative
15.6	4	Positive
11	5	Positive
10.5	4	Positive
14	7	Negative
16.3	5	Positive
15.1	5	Positive
17.1	7	Negative
13.8	7	Positive
17.1	7	Negative
16.7	7	Negative
17.6	7	Negative
16	7	Positive
15	7	Positive
18.1	7	Positive
17.5	7	Positive
18.2	6	Positive
17.7	7	Negative
16.7	7	Positive
16.8	7	Negative

Retention Time (min)	Retention Window	Polarity
16.7	2	Positive
17.6	2	Negative
4.5	4	Negative
17.5	3	Negative
16.4	3	Positive
16	13	Positive
3.4	3	Positive
18	5	Negative
16.3	4	Positive
11.7	15	Negative
16.1	3	Negative
17	5	Positive
23	6	Positive
17.2	13	Positive
15.9	4	Negative
16.5	5	Positive
16.8	5	Positive

15.6	3	Positive
13.2	3	Positive
15.6	3	Positive
11	3	Positive
10.5	4	Positive
15	6	Negative
18.1	4	Negative
15.8	13	Positive
16	5	Positive
17	6	Negative
17	4	Positive
5	4	Positive
14.6	2	Positive
5.7	3	Positive
2	3	Positive
17.2	2	Negative
12.3	3	Negative
17.5	4	Negative
19.2	5	Negative
16.4	4	Negative
16.6	4	Positive
10	8	Negative
16.5	5	Negative
17.7	3	Negative
13.3	3	Negative
12.5	5	Negative
12.5	8	Negative
12.4	4	Negative
16.6	4	Negative
15.5	4	Positive
16.3	4	Positive
17.7	5	Positive
16.5	4	Positive
16.2	4	Negative
15.4	4	Positive
7	5	Positive
10.9	5	Positive
17.5	9	Positive
12.5	4	Positive
14.9	4	Positive
15.3	3	Positive
4	3	Negative
15.5	6	Positive
13	5	Positive
16	4	Positive
6.6	4	Negative
16.5	6	Negative
12.1	5	Positive
2.4	4	Negative

9.7	4	Positive
6.6	8	Negative
11.4	4	Positive
23.3	5	Positive
15.5	6	Negative
12.6	4	Positive
13	5	Positive
4.8	3	Negative
15.3	4	Positive
10.3	9	Positive
15.7	4	Positive
23.5	6	Positive
3.7	5	Positive
23.4	4	Positive
2.4	3	Positive
4.4	5	Negative
12	4	Positive
22.7	4	Positive
4.8	3	Negative
9.2	6	Positive
18	3	Negative
14.3	4	Positive
3.2	3	Positive
3.5	4	Negative
16.2	2	Negative
16	8	Negative
3.6	6	Positive
17.9	4	Negative
14.4	4	Positive
22.3	11	Positive
15.6	4	Positive
2.4	3	Positive
14.3	5	Negative
15.7	3	Positive
10.5	4	Positive
13.8	4	Positive
15	5	Negative
4	5	Positive
12.2	4	Negative
4.1	4	Negative
13.7	4	Positive
3.9	4	Positive
