# Supplementary material for: β-cell–selective inhibition of DNA damage response signaling by nitric oxide is associated with an attenuation in glucose uptake
Source: J Biol Chem. 2023 Feb 10;299(3):102994. doi: 10.1016/j.jbc.2023.102994 (PMC10023961; doi:10.1016/j.jbc.2023.102994)
Supplement: Enzyme abbreviation [file mmc2.pdf]

Enzyme abbreviation

DDR signaling	
ATM	Ataxia-telangiectasia mutated protein
ATR	ATM- and Rad3-related protein
DNA-PK	DNA-dependent protein kinase
Glycolysis	
GK	Glucokinase
HK	Hexokinase
PGI	Phosphoglucose isomerase
PFK	Phosphofructokinase
TPI	Triosephosphate isomerase
GAPDH	Glyceraldehyde-3-phosphate dehydrogenase
PGK	Phosphoglycerate kinase
PGM	Phosphoglycerate mutase
PK	Pyruvate kinase
TCA cycle	
PDH	Pyruvate dehydrogenase
CS	Citrate synthase
ACO	Aconitase
IDH	Isocitrate dehydrogenase
αKDH	α-Ketoglutarate dehydrogenase
SCS	Succinyl-CoA synthetase

SDH	Succinate dehydrogenase
FUM	Fumarase
MDH	Malate dehydrogenase
Pentose phosphate pathway	
G6PD	Glucose-6-phosphate dehydrogenase
6PGD	6-Phosphogluconate dehydrogenase
RPI	Ribose-5-phosphate isomerase
RP3E	Ribulose-phosphate 3-epimerase
